# Experiences and perceptions of using a low-cost pet robot for older adults and people with dementia

**DOI:** 10.1093/geroni/igab046.3390

**Published:** 2021-12-17

**Authors:** Wei Qi Koh, Sally Whelan, Pascale Heins, Dympna Casey, Elaine Toomey, Rose-Marie Dröes

**Affiliations:** 1 National University of Ireland Galway, Galway, Galway, Ireland; 2 Maastricht University, Maastricht, Limburg, Netherlands; 3 University of Limerick, Limerick, Limerick, Ireland; 4 Vrije Universiteit Amsterdam (Amsterdam UMC), Amsterdam, Noord-Holland, Netherlands

## Abstract

Pet robots are a practicable substitute for animal-assisted therapy. They have been shown to have positive impacts on older adults, including people with dementia, such as providing companionship and facilitating social interaction. However, the issue of affordability can hinder equal access to such technology. The purpose of our study was to understand the perceptions and experiences of using a low-cost, commercially available pet robot with older adults and people with dementia. We used a novel methodology of analysing a large volume of user reviews that were collected from 15 consumer websites. A total of 1,327 user reviews that met our pre-specified inclusion criteria were included. Descriptive statistics was applied to characterise demographic data, and inductive qualitative content analysis was used to identify themes in the textual data. Most reviews were obtained from consumer sites in the United States, and most reviewers were family members of the users (i.e., older adults and people with dementia). We found that circumstantial reasons, such the inability to own live animals, prompted reviewers to purchase the pet robot. Most reviewers had positive perceptions of the pet robot, and described various activities that users engaged in with it. Impacts of using the pet robot, such as positive emotions, were also described. Finally, experiences about practical aspects of its use, such as durability and hygiene, were discussed. Overall, this study provides useful knowledge that can help researchers, robot developers and clinicians understand the viability of using low-cost pet robots to benefit older adults, including people with dementia.

